# Food-Intake Normalization of Dysregulated Fatty Acids in Women with Anorexia Nervosa

**DOI:** 10.3390/nu11092208

**Published:** 2019-09-13

**Authors:** Nhien Nguyen, Michelle Dow, Blake Woodside, J. Bruce German, Oswald Quehenberger, Pei-an Betty Shih

**Affiliations:** 1 Department of Psychiatry, School of Medicine University of California, San Diego, La Jolla, CA 92037, USA; n7nguyen@ucsd.edu; 2 Division of Medical Genetics, Department of Medicine, University of California, San Diego, La Jolla, CA 92093, USA; mdow@eng.ucsd.edu; 3 Department of Psychiatry, University of Toronto, Toronto, ON M5T 2S8, Canada; b.woodside@utoronto.ca; 4 Department of Food Science & Technology, University of California, Davis, Davis, CA 95616, USA; jbgerman@ucdavis.edu; 5 Department of Pharmacology, University of California, San Diego, San Diego, CA 92093, USA; oquehenberger@ucsd.edu

**Keywords:** fatty acids, anorexia nervosa, metabolic dysregulation

## Abstract

Anorexia nervosa (AN) is a psychiatric disorder affected by psychological, environmental, and biological factors. Individuals with AN avoid high-fat, high-calorie diets and have shown abnormal metabolism of fatty acids (FAs), which are essential for brain and cognitive/neuropsychiatric health. To clarify the relationship between FAs and AN, fasting and postprandial plasma FAs in AN patients and age-matched control women were analyzed via mass-spectrometry. Clinical phenotypes were assessed using Becker Anxiety Inventory and Becker Depression Inventory. AN patients and controls exhibited different FA signatures at both fasting and postprandial timepoints. Lauric acid, eicosapentaenoic acid (EPA), docosapentaenoic acid (DPA), and alpha-linoleic acid (ALA) were higher in AN than in controls (lauric acid: 15,081.6 ± 14,970.2 vs. 8257.4 ± 4740.2 pmol/mL; ALA at fasting: 2217.7 ± 1587.6 vs. 1087.9 ± 821.2 pmol/mL; ALA at postprandial: 1830.9 ± 1115.6 vs. 1159.4 ± 664.7 pmol/mL. EPA: 33,788.3 ± 17,487.5 vs. 22,860.6 ± 12,642.4 pmol/mL; DPA: 32,664.8 ± 16,215.0 vs. 20,969.0 ± 12,350.0 pmol/mL. FDR-adjusted *p*-values < 0.05). Food intake and AN status modified the correlations of FAs with body mass index (BMI), depression, and anxiety. Desaturases SCD-18 and D6D showed lower activities in AN compared to controls. Altered FA signature, specifically correlations between elevated *n*-3 FAs and worsened symptoms, illustrate metabolic underpinnings in AN. Future studies should investigate the mechanisms by which FA dysregulation, specifically elevated *n*-3 FAs, affects AN risk and outcome.

## 1. Introduction

Anorexia nervosa (AN) is an eating disorder characterized by distorted perceptions of the body image and extreme self-restraints on dietary intake to lose weight [1]. AN affects 0.3%–4% of women [2,3,4]. It is one of the deadliest psychiatric disorders with estimated mortality rates ranging between 4.4% and 18% [4,5]. Interactions between environmental, psychological, and biological factors have been proposed to affect the pathogenesis and treatment resistance characteristics in AN [2,6,7]. However, the biological etiology of AN remains elusive. Limited understanding of the AN pathophysiology has hindered the development of effective treatments and prevention strategies, as indicated by a high relapse rate of 11%–36% [8,9,10,11] and the persistence of eating disorder symptoms even after AN patients have restored their ideal weight after treatments [12]. Thus, expanded knowledge of the biological underpinnings of AN is required to enhance treatment effectiveness and reduce relapse. 

AN is characterized by distorted perceptions of the body and extreme self-restraints on dietary intake to lose weight [1]. Compared to healthy controls, women with AN display a strong aversion toward foods high in calories and fats [13,14] and prefer consuming diets containing low amounts of fats [15]. Reduced fat intake among AN patients, as a result of avoiding fat-rich foods, is concerning because lower amount of total lipids in the diet correlates with longer duration of the disorder [15]. AN patients with poor treatment outcomes have shown to consume less fat compared to AN patients with successful treatment outcomes [16], suggesting that fat restriction may be connected to metabolic changes that accelerate the pathogenesis of AN. Consistent with this concept, abnormal absorption and metabolism of lipids have been postulated to contribute to homeostatic disruptions that underlie AN pathophysiology [17]. Since fat aversion may impede treatment success and worsen AN prognosis, it is imperative to elucidate the biological changes related to the fat avoidance behavior to shed light on the etiology of AN and improve treatment outcomes.

AN patients avoid a broad range of high-fat foods, encompassing major sources of saturated and unsaturated fatty acids (FAs). While saturated FAs do not contain any double bond, unsaturated FAs contain one or more double bonds [18], which are termed monounsaturated and polyunsaturated FAs, respectively [19]. Unsaturated FAs can be classified into *n*-3, *n*-6, *n*-7, and *n*-9 FAs, which can be found in a variety of food staples including fish oil (*n*-3), soybean oil (*n*-6), butter (*n*-7), and olive oil (*n*-9) [20,21,22,23]. These FAs, especially the well-studied *n*-3 FAs, are important for brain development, and aberrations in *n*-3 FA levels have been implicated in various cognitive and neuropsychiatric disorders such as Alzheimer’s disease, autism, schizophrenia, anxiety, and depression [24,25,26]. Altered concentrations of FAs have been found in the blood samples of patients with AN in multiple studies. For instance, two studies, including our previous publication, reported significant increases in *n-*3 FAs in AN patients compared to controls [27,28]. However, two other studies found a decrease of long chain *n*-3 polyunsaturated FAs in AN and eating disorder patients [29,30]. A study found a significant decrease in the saturated stearic acid [31], whereas another study documented elevated saturated FAs in AN patients compared to healthy controls [28]. Abnormal patterns of FAs in AN, coupling to the documented importance of FAs in the development of a healthy and functional brain, suggest that lipid dysregulation is involved in the pathogenesis of AN. 

It is currently unknown if metabolic FA aberrations affect the pathophysiology of AN and if they could potentially serve as useful biomarkers for AN, as well as for assessing the outcome of treatment. Our study aims to replicate the previously found association [27] between plasma FAs and AN. Furthermore, we implemented a food-challenge study designed to assess if FA dysregulation is implicated in the fatty-food avoidance behavior frequently seen in AN. Lastly, to determine if FA metabolism is altered in AN, we compared the activity markers of enzymes regulating FA desaturation and elongation. We hypothesized that AN patients will show abnormal patterns of plasma FAs and altered enzyme activities demonstrating the presence of metabolic lipid dysregulation in both fasting and fed states, and that these dysregulation associate with clinical phenotypes. Answers to these questions will help the eating disorder field to better elucidate the biological underpinnings of AN, enable identification of novel biomarkers, and allow for more accurate prediction of treatment response as well as more effective prevention and intervention programs for AN. 

## 2. Subjects and Methods 

### 2.1. Participants and Study Flow

Participants were recruited from the University of Toronto and the University of California, San Diego (UCSD). A total of 50 women with AN (30 Ill AN (age 29.6 ± 9.0) and 20 Recovered AN (age 31.1 ± 12.0)) and 47 healthy control women (age 30.5 ± 9.3) enrolled in the study. At the fasting timepoint, blood samples and research questionnaires were collected from all participants. At the postprandial timepoint, blood samples and questionnaires were obtained from 28 women with AN (9 ill and 19 recovered AN) and 46 healthy control women. Prospective subjects who are interested in the study contacted recruitment site investigators and underwent a phone interview that assessed eligibility and received information about the study. Subjects with AN had to have a formal diagnosis with AN by a licensed psychiatrist. Exclusion criteria for all prospective participants included having an Axis I psychiatric illness, organic brain syndrome, schizophrenia or schizoaffective disorder, untreated thyroid disease, renal disease, hepatic disease, being pregnant or breast feeding, or using fish oil supplements on a regular basis. Subjects who reported regular use of fish oil supplements and were willing to discontinue fish oil supplementation for 30 days prior to the study visit remained eligible for the study if all other eligibility criteria have been met. Other supplements and/or medications were not included in the exclusion criteria. All study subjects fasted for a minimum of 10 h before their scheduled study visit time. Upon arriving at the clinic, study participants completed the consent form and questionnaires and donated blood, and all participants ate a breakfast sandwich as the study challenge meal that consists of sausage, egg, cheese, and English muffin (Calories: 436; fat: 27 g (saturated fat = 11 g); carbohydrates: 28 g; protein: 19 g). All participants finished the meal within 25 min. Two hours after eating, all subjects donated another blood sample and completed postprandial questionnaires. Although there were similar numbers of individuals with AN (*n* = 50) and healthy controls (*n* = 47) at the fasting timepoint, 22 subjects in the AN group and 1 subject in the control group had refused the challenge meal due to reasons including “too sick to eat” (*n* = 13, all AN) and refusal to eat any meat or pork product (*n* = 9). This study has been approved both by the UCSD Human Protection Board and the University of Toronto Research Ethics Board.

### 2.2. Fatty Acid Analysis

The plasma FA concentrations were measured by gas chromatography-mass spectrometry (GC-MS) at the UCSD Lipidomics Core as previously reported [27,32,33]. Briefly, to analyze the total (sum total of esterified and non-esterified) plasma FA composition, human plasma (10 μL) in methanol (250 μL) was supplemented with a cocktail of internal standards consisting of 15 deuterated FAs, saponified with 0.5 N KOH for 30 min at 37 °C and then adjusted to pH = 4 with glycine buffer. The FAs were extracted with isooctane and derivatized with pentafluorobenzyl (PFB) bromide. The resulting fatty acid PFB esters were analyzed by GC-MS using a negative chemical ionization mode (Agilent 6890N gas chromatograph equipped with an Agilent 5973 mass selective detector; Agilent, Santa Clara, CA, USA). All reagents and solvents were of highest purity, suitable for mass spectral analyses and were purchased from ThermoFisher Scientific (Waltham, MA, USA). All fatty acid standards (purity > 99%) used for identification and quantification were purchased from Nu-Chek Prep Inc. (Elysian, MN, USA). All deuterated fatty acids that were used as internal standards were purchased from Cayman Chemical (Ann Arbor, MN, USA). Standard curves for each of the FAs were acquired in parallel using identical conditions. The quantitative assessment of FAs in a sample was achieved by comparison of the mass spectrometric ion signal of the target molecule normalized to the internal standard with the matching standard curve according to the isotope dilution method. The concentrations of the FAs are reported in pmol/mL. 

### 2.3. Enzyme Activity Indices

The following product/substrate FA ratios were used as proxy markers of a number of desaturase and elongase activities: For stearoyl-CoA desaturase-16 (SCD-16): palmitoleic acid/palmitic acid [34,35,36]; for stearoyl-CoA desaturase-18 (SCD-18): oleic acid/stearic acid [34,36]; for delta-5-desaturase (D5D): arachidonic acid (ARA)/dihomo-gamma-linoleic acid (DGLA) [34,35,36]; for delta-6-desaturase (D6D): stearidonic acid/ALA [37], gamma-linoleic acid (GLA)/linoleic acid (LA) [34,35], and DGLA/LA [36]; for elongase 2 (ELOVL2): adrenic acid/ARA [38]; for elongase 5 (ELOVL5): DGLA/GLA [34]; and for elongase 6 (ELOVL6): stearic acid/palmitic acid [34].

### 2.4. Statistical Analysis

Phenotype data (age, body mass index (BMI), Becker depression inventory (BDI), and Becker anxiety inventory (BAI)) were tested for normality using histograms and Shapiro-Wilk test of normality in R 3.5.2. Wilcoxon rank sum was used to compare study subject characteristics and psychiatric phenotypes between control group and each of the AN groups (ill AN (IAN), recovered AN (RecAN), all AN combined (all AN)) (Table 1). We evaluated 26 FAs in the *n*-3, *n*-6, *n*-7, *n*-9, and saturated FA classes in all available participants at fasting and postprandial timepoints. The pairwise similarity was determined using the Pearson correlation between absolute FA concentrations and plotted using seaborn clustermap in Python with row or column clustering set to False. Python was used to run *t*-tests with false discovery rate (FDR) adjustment to compare plasma concentrations of 26 FAs in AN versus controls at both fasting and postprandial timepoints. Spearman’s rho was used to determine correlations between individual FAs and phenotypes (BMI, BDI, BAI, and postprandial change in BAI). FA product/substrate ratios were generated as proxy markers of FA regulatory enzyme activities. *T*-test was used to compare FA product/substrate ratios as well as *n*-6:*n*-3 ratios between AN and controls.

## 3. Results

### 3.1. Participant Characteristics and Phenotypes

No significant difference in age was found between AN and healthy controls (*p*-value = 0.88) (Table 1). The mean BMI in controls was 21% higher than the mean BMI in all AN subjects (22.9 ± 3.5 vs. 18.9 ± 3.8, respectively; *p*-value < 0.01), 37% higher than ill AN (IAN) subjects (22.9 ± 3.5 vs. 16.7 ± 2.4, *p*-value < 0.001) and 3% higher than recovered AN (RecAN) subjects (22.9 ± 3.5 vs. 22.3 ± 2.8, *p*-value = 0.5) (Table 1). Depression score was 4.6 times higher in all AN than that in controls (21.8 ± 16.4 vs. 3.9 ± 7.4; *p*-value < 0.001). When stratified by AN recovery status, depression was more than 6 times higher in IAN and 2 times higher in RecAN compared to controls (IAN: 28.0 ± 16.7; RecAN: 12.6 ± 10.9; controls: 3.9 ± 7.4; *p*-values < 0.001). At the fasting timepoint, mean anxiety score was 4 times higher in all AN compared to controls (21.5 ± 13.7 vs. 4.3 ± 5.8; *p*-value < 0.001). The IAN group exhibited 5.2 times higher anxiety score, while RecAN group had 2.3 times higher anxiety score than that of controls (IAN: 26.6 ± 13.1; RecAN: 14.4 ± 11.6; controls: 4.3 ± 5.8; *p*-values < 0.001) (Table 1). All AN and RecAN groups showed greater reductions in anxiety score two hours after eating the meal when compared to controls (*p*-values = 0.013 (All AN) and 0.025 (RecAN), respectively, Table 1). 

### 3.2. Fatty Acid Profile

**Figure 1:**
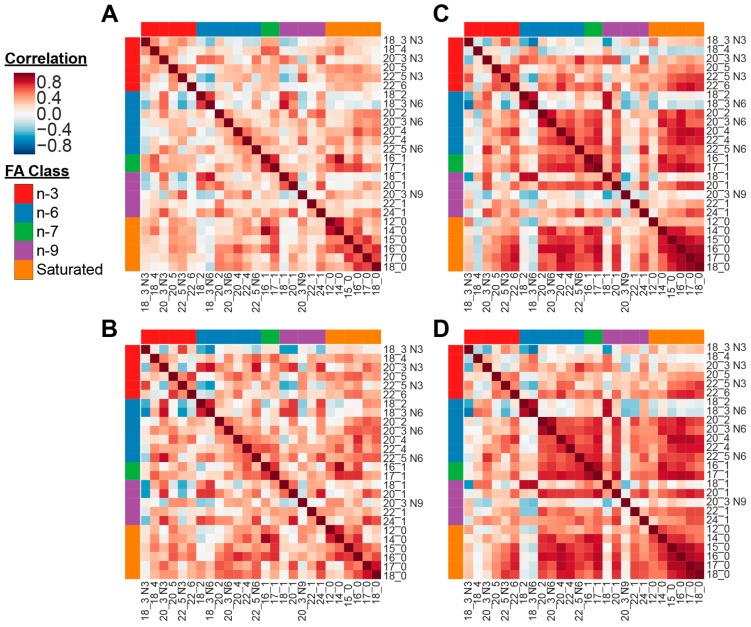
Heatmaps representing pairwise correlations between all fatty acids in anorexia nervosa (**A** (fasting) and **B** (postprandial)) and healthy controls (**C** (fasting) and **D** (postprandial)). Fatty acid classes (*n*-3, *n*-6, *n*-7, *n*-9, and Saturated) are indicated by the color bars on the top and left side of the heatmaps. In the heatmap, red represented higher correlation, and blue shows lower correlation between a pair of markers. (A) AN group at fasting timepoint. (B) AN group at postprandial timepoint. (C) Control group at fasting timepoint. (D) Control group at postprandial timepoint.

At both fasting and postprandial timepoints, more positive correlations were observed among the 6 saturated and 20 unsaturated FAs in healthy controls than in AN patients, suggesting that FA metabolism is altered in AN in both fasting and fed states (Figure 1). The correlation patterns show distinguishable differences in FA concentrations between AN (Figure 1A,B) and controls (Figure 1C,D) across the two time points. At both fasting and postprandial timepoints, controls exhibited stronger positive correlations between *n*-6 and *n*-7 FAs when compared to AN. Similarly, subgroups of correlations between *n*-6, *n*-7 and saturated FAs were observed in controls but not in AN. Within the AN group, the correlations between the FAs generally became more pronounced after eating (Figure 1A vs. Figure 1B). AN showed stronger correlations between *n*-6 and *n*-7, as well as *n*-6 and saturated FAs at the postprandial timepoint compared to the fasting state. In the control group, FA correlations were similar between fasting and postprandial timepoints (Figure 1C vs. Figure 1D), with some stronger/more positive correlations between *n*-6, *n*-7, and *n*-9, as well as between *n*-9 and saturated FAs in the postprandial state. 

### 3.3. Fasting and Postprandial Concentrations of Individual Fatty Acids

**Figure 2:**
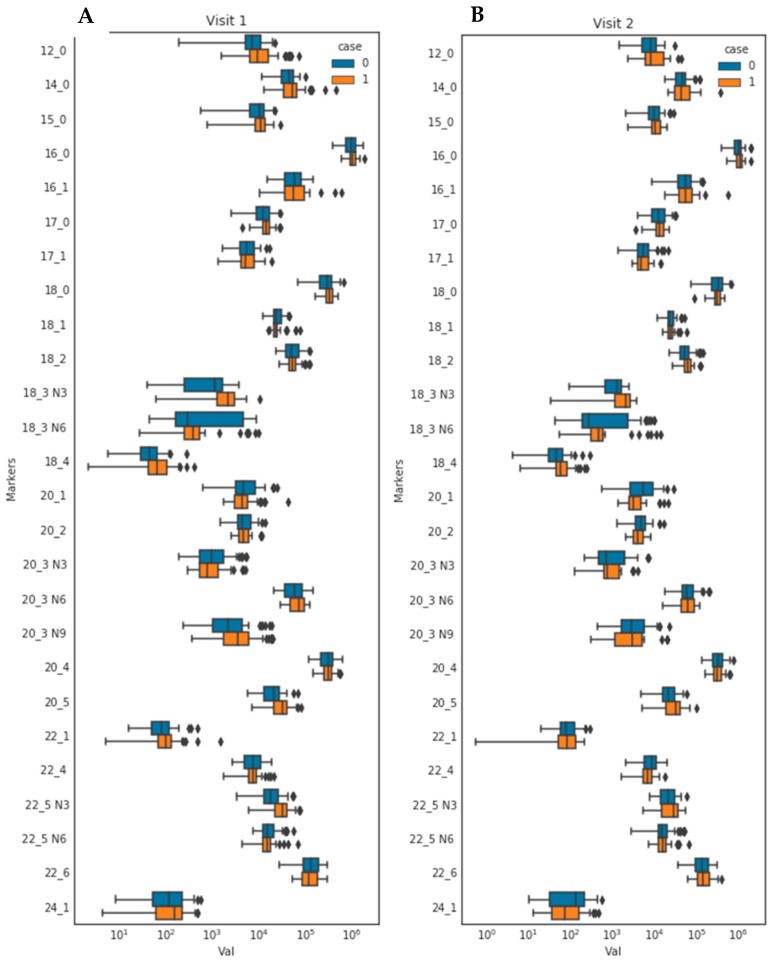
Boxplots representing distribution of each fatty acid in anorexia nervosa group (Orange) and control group (Blue). Orange bars: AN group; blue bars: control group. Visit 1 (**A**) refers to the fasting timepoint, Visit 2 (**B**) refers to the postprandial timepoint (two hours after eating a standardized sandwich). The pairwise similarity was determined using the Pearson correlation between FA concentrations and plotted using seaborn clustermap in Python. *T*-test with false discovery rate (FDR) adjustment was used to determine statistical differences between AN and control groups for each individual fatty acid.

Out of the 26 FAs examined, four (one saturated and three *n*-3 FAs) at fasting visit and one (*n*-3 FA) showed significant increases in AN patients when compared to healthy controls after FDR corrections (Figure 2A). At fasting timepoint, the saturated lauric acid (12:0) concentration in AN was 1.8 times that in controls (mean ± SD: 15,081.6 ± 14,970.2 pmol/mL and 8257.4 ± 4740.2 pmol/mL, respectively; *p*-value = 0.004; FDR-adjusted *p*-value = 0.023), while the n-3 polyunsaturated alpha-linolenic acid (18:3 *n*-3, ALA) in AN was twice the concentration of that in controls (2,217.7 ± 1587.6 pmol/mL vs. 1087.9 ± 821.2 pmol/mL; *p*-value < 0.0001; FDR-adjusted *p*-value = 0.0009) (Figure 2A). Eicosapentaenoic acid (20:5 *n*-3, EPA) and docosapentaenoic acid (22:5 *n*-3, DPA) concentrations among AN were 48% and 56% higher than those in controls (EPA: 33,788.3 ± 17,487.5 pmol/mL vs. 22,860.6 ± 12,642.4 pmol/mL; *p*-value = 0.0007; FDR-adjusted *p*-value = 0.006. DPA: 32,664.8 ± 16,215.0 pmol/mL vs. 20,969.0 ± 12,350.0 pmol/mL; *p*-value = 0.0001; FDR-adjusted *p*-value = 0.002) (Figure 2A). At the postprandial state, the mean ALA concentration in AN was 1.6 times that of healthy controls (1,830.9 ± 1115.6 pmol/mL vs. 1159.4 ± 664.7 pmol/mL; *p*-value = 0.002; FDR-adjusted *p*-value = 0.046) (Figure 2B). 

### 3.4. Fatty Acid Correlations with Body Mass Index and Psychiatric Phenotypes

The correlations between FAs and AN phenotypes (BMI, depression, fasting anxiety, and change in anxiety after eating) are shown in Table 2 for the fasting timepoint, and Table 3 for the postprandial timepoint. In all subjects, elevation in all four FAs were significantly correlated with lower BMI in fasting state (Lauric acid: Spearman’s rho *r*_s_ = −0.28; *p*-value = 0.005. ALA: *r*_s_ = −0.46, *p*-value < 0.01. EPA: *r*_s_ = −0.29, *p*-value = 0.004. DPA: *r*_s_ = −0.39, *p*-value < 0.01) (Table 2). Increased concentrations of fasting *n*-3 ALA, EPA, and DPA were also significantly associated with higher levels of depression (BDI score) (ALA: *r*_s_ = 0.31; *p*-value = 0.002. EPA: *r*_s_ = 0.28; *p*-value = 0.005. DPA: *r*_s_ = 0.31; *p*-value = 0.002) and higher levels of anxiety (BAI score) (ALA: *r*_s_ = 0.28; *p*-value = 0.006. EPA: *r*_s_ = 0.26; *p*-value = 0.01. DPA: *r*_s_ = 0.24; *p*-value = 0.019) (Table 2). The saturated lauric acid showed no significant correlation with any phenotypes. None of the four FAs were associated with postprandial change in BAI score (Table 2). At the postprandial state, significant inverse associations with BMI remained for lauric acid and ALA, while significant correlations with BDI and BAI also held true for ALA and EPA. A higher concentration of postprandial ALA was associated with a smaller decrease of post-meal anxiety (*r*_s_ = −0.25; *p*-value = 0.03) (Table 3).

In AN only, lower BMI was significantly correlated with higher fasting *n*-3 ALA and DPA (ALA: *r*_s_ = −0.33; *p*-value = 0.019. DPA: *r*_s_ = −0.3; *p*-value = 0.032). On the contrary, controls exhibited no significant correlations between BMI and any of the four FAs (Table 2). In both AN and control groups, no significant associations between FAs were found with fasting depression, fasting anxiety, or postprandial change in anxiety (Table 2). At postprandial timepoint, higher lauric acid was inversely correlated with lower BMI (*r*_s_ = −0.36; *p* = 0.058) in AN but not in controls (*p*-value > 0.05) (Table 3). Increased postprandial concentrations of ALA and EPA were significantly associated with higher postprandial level of anxiety in controls (ALA: *r*_s_ = 0.34; *p*-value = 0.022. EPA: *r*_s_ = 0.33; *p*-value = 0.026) but not in the AN (*p*-values > 0.05) (Table 3). 

### 3.5. Desaturase and Elongase Indices

**Figure 3:**
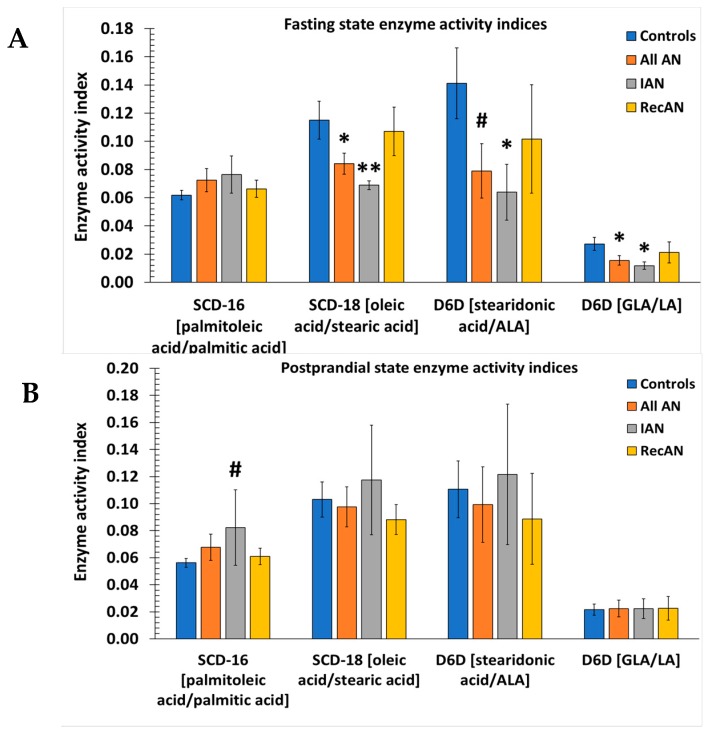
Markers of desaturase and elongase indices associated with AN status. Fasting (**A**) and postprandial (**B**) enzyme activity indices estimated by fatty acid ratios. Entries are ratios formed by concentrations of individual fatty acids. Fatty acid has unit of measurement of pmol/mL. Bars and error bars represent mean and standard error of the mean. *T*-test was used to compare mean ratio markers for each individual group of AN (All AN, ill AN, recovered AN) with controls. Statistics: # = 0.05 ≤ *p*-value < 0.10; * = 0.01 ≤ *p*-value < 0.05; ** = *p*-value < 0.01. LA: linoleic acid; ALA: alpha-linoleic acid; GLA: γ-linolenic acid. IAN: Ill anorexia nervosa; RecAN: recovered anorexia nervosa. SCD-16: stearoyl-CoA desaturase-16 (palmitoleic acid/palmitic acid); SCD-18: stearoyl-CoA desaturase-18 (oleic acid/stearic acid); D6D: delta-6-desaturase (for *n*-3: stearidonic acid/ALA; for *n*-6: GLA/LA).

Product/precursor ratios of FAs are proxy markers of enzyme activities such as SCD-16 and 18, D5D, D6D, and ELOVL 2, 5, and 6. These ratio markers were used to estimate the in vivo activities of desaturases and elongases and compared between AN and controls to explore whether FA regulatory enzymes contribute to FA differences found in AN. In the fasting state, three proxy markers were significantly different between AN group and control group (Figure 3A). The marker for SCD-18 and two markers for D6D (represented by *n*-3 FAs and *n*-6 FAs) were decreased in all AN compared to controls (SCD-18: AN: 0.08 vs. controls: 0.12; *p*-value = 0.04; D6D (*n*-3): AN: 0.08 vs. controls: 0.14; *p*-value = 0.05; D6D (*n*-6): AN: 0.02 vs. controls: 0.03; *p*-value = 0.04). Comparison of IAN and control groups showed a more significant reduction of SCD-18 in IAN (0.07 vs. 0.12; *p*-value = 0.008 (Figure 3A). In the fed state, none of the three activity markers remained significantly different between AN and controls. However, SCD-16 index was noticeably higher in IAN compared to controls (IAN: 0.08 vs. controls: 0.06; *p*-value = 0.07) (Figure 3B).

### 3.6. n-6 to n-3 Fatty Acid Ratios

**Figure 4:**
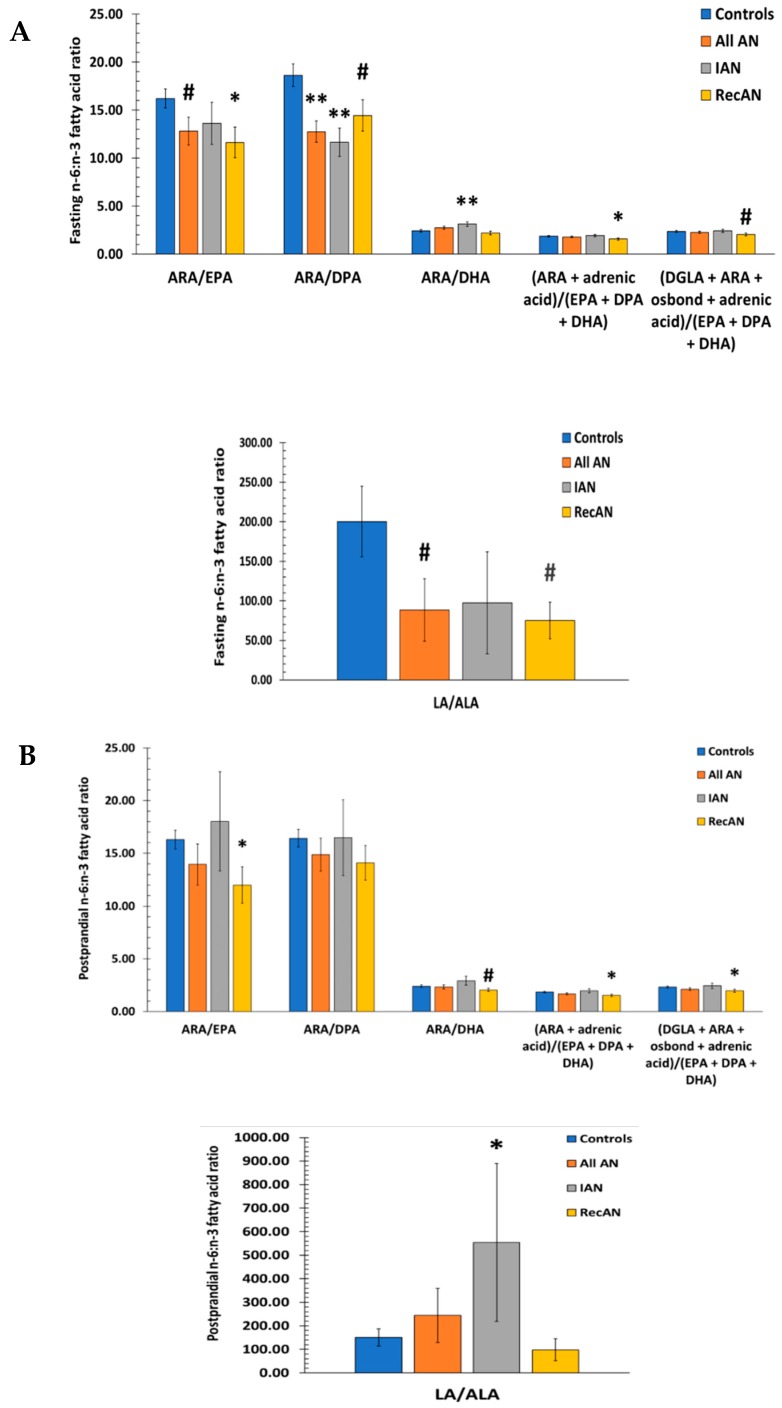
Fasting (**A**) and postprandial (**B**) *n*-6:*n*-3 fatty acid ratios. Entries are ratios formed by individual fatty acids. Fatty acid has unit of measurement of pmol/mL Bars and error bars represent mean and standard error of the mean. T-test was used to compare mean ratio for each individual group of AN (all AN, ill AN, recovered AN) with controls. Statistics: # = 0.05 ≤ *p*-value < 0.10; * = 0.01 ≤ *p*-value < 0.05; ** = *p*-value < 0.01. LA: linoleic acid; ALA: alpha-linolenic acid; ARA: arachidonic acid; EPA: eicosapentaenoic acid; DPA: docosapentaenoic acid; DHA: docosahexaenoic acid. DGLA: Dihomo-γ-linolenic acid. IAN: Ill anorexia nervosa; RecAN: recovered anorexia nervosa.

Ratios between well-studied *n*-6 and *n*-3 FAs were calculated to examine the relationship between *n*-6:*n*-3 ratio and AN risk. Linoleic acid (18:2, *n*-6 LA): ALA ratio decreased by 56% in all AN compared to controls during fasting (*p*-value = 0.06) (Figure 4A) but was not statistically different in the fed state (*p*-value = 0.36, Figure 4B). Compared to controls, RecAN showed a 63% decrease in LA:ALA (*p*-value = 0.08) during fasting (Figure 4A), but not in postprandial state (*p*-value = 0.41) (Figure 4B). In contrast, IAN showed an 267% increase in postprandial LA:ALA compared to controls (*p*-value = 0.018) (Figure 4B). The ratio of arachidonic acid (20:4, *n*-6 ARA) to EPA was marginally lower in all AN during fasting (*p*-value = 0.06) (Figure 4A). In RecAN however, ARA:EPA ratio was significantly reduced at both fasting (by 28%, *p*-value = 0.015) and postprandial (by 26% *p*-value = 0.019) states (Figure 4). ARA:DPA ratio in all AN was significantly lower than that in controls during fasting (by 32%, *p*-value < 0.001) but not after eating (*p*-value = 0.34) (Figure 4). ARA:DPA was decreased by 37% in IAN (*p*-value < 0.001) and by 23% in RecAN (*p*-value = 0.051) during fasting (Figure 4A).

The ARA to docosahexaenoic acid ratio (22:6, *n*-3 DHA), (ARA + adrenic acid): (EPA + DPA + DHA) ratio, and (DGLA + ARA + osbond + adrenic acid): (EPA + DPA + DHA) ratio were not significantly different between AN and controls in either fasting or postprandial states. In IAN only, ARA:DHA was increased by 29% (*p*-value = 0.004) in the fasting state whereas in RecAN, (ARA + adrenic acid): (EPA + DPA + DHA) was significantly lowered by 15% at fasting (*p*-value = 0.041) and by 17% in postprandial (*p*-value = 0.022) states (Figure 4). (DGLA + ARA + osbond + adrenic acid): (EPA + DPA + DHA) ratio was 13% lower in RecAN at fasting (*p*-value = 0.081) and 16% lower in postprandial (*p*-value = 0.037) states (Figure 4).

## 4. Discussion

Fatty acids (FAs) have been reported to be dysregulated in anorexia nervosa (AN) in several studies albeit of modest sample sizes [27,28,31]. To explore the role of FAs in the pathogenesis of AN, we examined the plasma concentrations of 26 well-known saturated and unsaturated FAs in an independent sample of women with AN and healthy controls during both fasting and fed metabolic states. These 26 FAs exhibited distinct patterns of correlations in AN compared to controls at both the fasting and postprandial timepoints (Figure 1), suggesting that AN patients have a FA profile indicative of dysregulated lipid metabolism. The differential relationships among FAs in AN patients in comparison to controls, along with abnormal concentrations of several FAs in AN, indicate that metabolic aberrations, namely lipid dysregulation, play a role in modulating the risk and symptoms of AN. Saturated FAs and unsaturated *n*-6 and *n*-7 FAs were more strongly correlated in controls compared to AN at both timepoints, indicating that metabolic networks are stimulated in AN that diminish these correlations and may contribute to AN pathogenesis. Controls showed generally similar FA patterns between the fasting and postprandial timepoints, with the exception of stronger correlations among *n*-6, *n*-7, *n*-9, and saturated FAs in the fed state. Meanwhile, AN had a visually striking differential FA pattern during fasting state but exhibited a more similar FA pattern to that of controls after eating, implying that food intake may have a normalizing effect on lipid dysregulation. This finding is encouraging particularly after consumption of just one modest calorie food item. 

The metabolic etiology of AN has been suggested by the identification of significant genetic correlations between AN-associated variants and various metabolic features (e.g., insulin, insulin resistance, type II diabetes, and obesity) in recent consortium studies [39,40]. A meta-analysis found that individuals with AN had elevated insulin sensitivity [41], further emphasizes a link between AN andmetabolic disorders. The involvement of metabolic factors in AN, especially those related to lipid regulation such as FA concentrations, suggests that studying factors that regulate FA synthesis and metabolism in AN may help to uncover the biological underpinnings of this illness. 

In the fasting state, three of the four significantly elevated FAs in AN belong to the *n*-3 polyunsaturated family (Figure 2A), suggesting that lipid dysregulation in AN can be mostly attributed to relative increases in *n*-3 polyunsaturated FAs. The *n*-3 FAs are derived either from the diet or by elongation of ALA. The dietary behavior practiced by AN patients may explain in part the disproportionately high *n*-3 FA intake through large amounts of leafy vegetables and quantitative down-regulation of lipid metabolism that is naturally low in *n*-3 polyunsaturated FAs. ALA can be elongated to 20:3 and 22:3; subsequent desaturation then produces 20:5, 22:5, and 22:6. Thus, metabolic compensatory mechanism may include differences in desaturase and elongase activities. The FAs in plasma lipoproteins are derived either from the diet or from fat stores. Both pathways converge in the liver where the FAs from either the chylomicrons or directly from the adipose tissue are repacked and secreted as very low-density lipoprotein (VLDL). Our data clearly show the FA differences to be more pronounced in the fasting state, which would suggest the involvement of the adipocytes. Enzymes that regulate the adipose FAs are lipoprotein lipase (LPL), which is the rate-limiting enzyme for the import of FAs into adipocytes [42,43,44]. Additionally, hormone-sensitive lipase (HSL) and adipocyte triglyceride lipase (ATGL) can hydrolyze the stored triacylglycerols (TAGs) and provide the liver with free FAs [45,46]. The activity or substrate specificity of any of these enzymes could be altered in AN, which ultimately would translate into changes in the FA composition of lipoproteins in circulation. While the differences in intestinal absorption of FAs due to differences in pancreatic lipase activity and host genetic factors may also alter circulating FAs, our finding that postprandial FA profile was partially normalized after eating a meal suggests that intestinal absorption may not be a key factor. Further studies are required to determine relevant mechanisms underlying the elevation of *n*-3 FAs found in AN, and whether increases in *n*-3 FAs drive lipid dysregulation to adversely affect the symptoms of AN, as opposed to other diseases (e.g., fatty liver, cardiovascular disease, and metabolic syndrome) where *n*-3 FA concentrations and supplementation have delivered generally positive effects [47,48,49,50]. 

The significantly higher fasting ALA and EPA concentrations in AN (Figure 2A) concur with our previous finding [27] and other studies [51], confirming the elevation of ALA and EPA in patients with eating disorders. The significantly increased fasting lauric acid, ALA, EPA, and DPA in AN (Figure 2A) are counterintuitive considering the implications that these FAs are crucial for a healthy brain, and may protect against several metabolic, inflammatory, and psychiatric disorders. For example, lauric acid has been shown to promote brain health by inducing formation of ketone bodies, which serve as alternative fuel for the brain in response to impaired glucose metabolism [52]. Lauric acid exerts both antibacterial and anti-inflammatory properties as it hindered *Clostridium difficile* infection and synthesis of inflammatory cytokines in mice [53]. Extant research has suggested that ALA may protect against coronary heart disease and stroke [47,48,49] and diminish autism-like phenotypes in rats [54]. EPA and DPA have been shown to be beneficial to brain health and neurodegenerative diseases [55] and protect against metabolic syndrome: high EPA and DPA were linked to 33% and 35% reduced risk of metabolic syndrome, respectively [50]. For medical populations that suffer from significant weight-loss like cancer patients, EPA has also been shown to enhance caloric intake and attenuate weight loss [56,57]. EPA supplementation was associated with weight gain and higher lean body mass in pancreatic cancer patients with a serious muscle wasting syndrome known as cachexia [58]. In a meta-analysis, adjunctive treatment with *n*-3 supplements containing ALA, EPA, or EPA and DHA have ameliorated depressive symptoms in bipolar disorder [59]. In schizophrenia, while the use of EPA-containing supplement in combination with antipsychotics did not confer significant benefit compared to standard antipsychotic monotherapy [60], *n*-3 supplementation has been shown to provide neuroprotective benefits to halt the development of psychotic disorders in ultra-high-risk individuals [61]. Given the generally beneficial effects these FAs show in other medical or psychiatric disorders, the relative elevation of these FAs in AN compared to healthy controls appears to be paradoxical. 

In eating disorders, studies of modest sample size showed that patients taking *n*-3 FA supplements during treatment exhibited improvements in body weight [51]. In addition, data from an open-case study and a case report showed that EPA supplementation as an adjunct therapy may boost weight gain in AN [62,63]. These findings contrast against the inverse correlations of BMI with ALA in women with AN in one study [64], and with inverse BMI correlations with both ALA and DPA observed in AN in this study (Table 2). Here, we replicated the results in our 2016 report [27] using a stricter, experimentally-controlled study design and show again that fasting *n*-3 FAs are elevated in AN. At the postprandial timepoint, dysregulated FAs have partly normalized and were no longer significantly different from concentrations observed in healthy controls with the exception of ALA (Figure 2B), suggesting that a single meal can “activate” lipid metabolism in AN and normalize the differential FA signature observed in the fasting state. Together, these data demonstrate that clinical improvements observed in earlier eating disorder studies may not result from only increased circulating *n*-3 FAs, but also through synergistic effects with treatment or total calorie increases during treatment sessions. Hence, more studies are required to clarify the mechanism by which *n*-3 dysregulation affects AN, and to clarify how diet and standard treatments synergistically normalize this dysregulation to enhance positive AN outcome.

Previous research has documented higher *n*-6:*n*-3 FA ratios in psychiatric disorders [65,66,67] and in comorbid depression among girls with eating disorders [68]. By contrast, a recent meta-analysis found that patients with eating disorders have a reduced ratio of *n*-6:*n*-3 FAs compared to healthy controls [51], which is consistent with our previous [27] and current findings. In the present study, AN patients showed significant decreases in fasting LA:ALA, ARA:EPA, and ARA:DPA ratios (Figure 4A) compared to controls. The majority of the *n*-6: *n*-3 FA ratios also showed significant or marginally significant decreases in IAN and RecAN compared to controls (Figure 4). These results indicate that decreased *n*-6:*n*-3 ratios in eating disorders and AN are driven in part by the abnormally high *n*-3 FA concentrations.

In our combined analysis, increases in fasting concentrations of the four differential FAs were significantly associated with lower BMI (Table 2), suggesting that these FAs may protect against obesity and contribute to the low body weight that individuals with AN exhibit. These results are consistent with the literature showing higher ALA concentration associating with a smaller increase in BMI in children 5–12 years old [69], yet another study did not find lauric acid consumption to be associated with BMI in adults [70]. Depression and anxiety are common mental health issues in the general population and are intensified in AN. Depression and anxiety levels were positively correlated with ALA, EPA, and DPA, indicating that higher concentrations of these FAs and/or the metabolic pathways that preferentially utilize them may be linked to changes in depression and anxiety. While one study reported increased ALA and EPA concentrations were associated with less depression and suicide attempts [71], a meta-analysis showed that eating disorder patients supplemented with *n*-3 FAs (mainly EPA and/or DHA) did not show improvements in mood symptoms [51]. Contradicting results may be attributed to heterogeneity in study subject characteristics and sample sizes, differences in assessment tools, and discrepancies in the dosage and administration period of *n*-3 supplementation across different studies. 

When stratified, it becomes evident that the inverse association with BMI was driven by the AN group. AN exhibited significant negative correlations of BMI with ALA and DPA while no significant BMI correlation was observed in the control group (Table 2). Both non-stratified and stratified associations illustrated differences in correlations between fasting and postprandial timepoints (Table 2 and Table 3), implicating that food intake modulates the relationships between FA concentrations and BMI, depression, and anxiety. After eating, ALA and EPA levels were significantly associated with higher postprandial anxiety in controls but not in women with AN (Table 3), which may be explained in part by a larger decrease of postprandial anxiety found in AN (Table 1). 

Product/precursor ratios of FAs were used as proxy markers of in vivo desaturases and elongases activities. Markers of SCD-16 and 18, D5D, D6D, and ELOVL 2, 5, and 6 were compared between AN patients and controls to explore whether regulatory enzymes are responsible for FA differences found in AN. Proxy markers of SCD-18 and D6D were lower in all AN patients compared to controls at fasting but not at postprandial states (Figure 3), suggesting that these FA desaturases and elongases may directly contribute to the differential FA signature observed in AN. The SCD-18 catalyzes the biosynthesis of the monounsaturated 18:1 oleic acid from the saturated 18:0 stearic acid, thus playing an important role in *de novo* lipogenesis as well as energy/lipid storage and metabolism [72]. The observed reduction in SCD-18 activity index among AN is consistent with previous findings that lower SCD-18 index (as well as SCD-16 index) correlated with lower body fat and BMI in older individuals [73], and that downregulation of SCD-1 (the collective name referring to both SCD-16 and SCD-18) hindered adiposity and obesity in leptin-deficient obese mice [74]. However, the present study’s findings on SCD are not consistent with reports that females with eating disorders had significantly elevated SCD-16 and SCD-18 indices [30,75,76]. Altogether, these observations imply that the effect of SCD on body weight may be disease-specific and population-dependent. D6D catalyzes the conversion of dietary ALA and LA into other *n*-3 and *n*-6 FAs, thus serve as key mediator of *n*-3 and *n*-6 FAs [77]. Similar to our result, a decrease in D6D activity has been reported in girls with eating disorders in comparison to controls [30]. While in another study, teenage girls with eating disorders exhibited increased D5D activity index [30,76] but in another study of adult women, AN patients exhibited lower D5D index compared to controls [75]. These mixed results demonstrate a need to further research the roles SCD-16, D5D, and D6D activities play in AN risk and metabolic dysregulation. 

A major strength of this study was data validation by replication of our earlier published results [27]. We have chosen well-matched AN patients and controls for this work (Table 1), which ensured that observed differences between the two groups were not due to common confounding effects. Additionally, we were able to tightly control the study protocol to capture both fasting and postprandial measurements to assess FA changes resulting from a controlled food intake. We show for the first time that food consumption normalized some of the relative differences in FA concentrations between AN patients and controls. In addition, food consumption altered relationships between fasting FA concentrations and AN phenotypes (BMI, depression, and anxiety levels). This study applied multiple comparison correction, successfully replicated our prior study findings, and enabled visualization of relationships amongst all FAs detected using mass-spectrometry lipidomics, thus providing a comprehensive picture of FA signature in AN. Lastly, a standardized sandwich was distributed to all participants in our study to keep the number of calories and fats consistent, thus eliminating the possibility that observed differences between AN patients and controls in the fed state resulted from variations in the meal. 

The present study was limited by the use of FA ratios as proxy markers of enzyme activities instead of direct measurements of these enzyme activities. It would have been ideal if the AN group (*n* = 50) and control group (*n* = 47) had similar sample sizes in both timepoints, however we had fewer AN (*n* = 28) consumed the meal due to patients’ refusal. The reduced sample size in the postprandial timepoint lowered the statistical power in the fed state analyses. Lastly, since cause-and-effect relationships could not be easily determined, it is unclear whether dysregulated fasting FA concentrations precede or follow/develop from the AN onset. 

This study did not take multiple postprandial timepoint measurements as this would add another layer of complexity to patient recruitment and analysis. A comprehensive assessment of time-dependent lipid metabolism as independent variables is complicated because individuals with AN have shown delayed gastric emptying [78,79,80,81,82,83] and intestinal transit [84,85]. Furthermore, the complex dynamics of gastrointestinal transit, nutrient uptake and post-prandial metabolism are highly variable across the human population. Discouragingly, relatively few studies have been conducted to describe the full range of human diversity, much less assign causal mechanisms or quantitative diagnostic protocols. Nonetheless, indications to date argue compellingly that variation in gastrointestinal dynamics is attributable in part to genetics [86], diet [87], infection [88], and of course the now ubiquitous microbiome [89]. Not surprisingly, altered intestinal transit dynamics have also been shown in a wide range of clinical conditions, some of which are also responsive to therapeutic intervention. We acknowledge that our choice to pick a single postprandial timepoint has the potential to introduce bias into the mechanisms behind our interventions. However, at this stage of the research it is neither possible to assign intestinal transit time as a discrete variable nor feasible to accurately capture the full range of inter-individual variation. 

FA signatures both at fasting and postprandial states were visually different in AN compared to controls, espousing the metabolic dysfunctions known in AN. Our data showed that eating a high-fat sandwich appears to normalize these dysregulations, as evidenced by reduced between-group differences in FA concentrations and desaturation activities. Saturated and *n*-9, *n*-7, *n-*6, and *n*-3 FAs are synthesized by ostensibly all higher organisms including humans. The implications of these FAs to whole body metabolism and especially to fuel regulation were first eloquently demonstrated by Cao et al. [90]. The source of these FAs could be the diet, de novo synthesis, or mobilization from adipose or tissue stores. While it is not possible to know which specific factor is most important in driving the changes observed in AN without exhaustive isotope enrichment studies, the correlations and associations identified enable us to enrich or deplete these FAs using dietary, pharmacologic or even microbial protocols to more precisely address mechanisms of action and therapeutic efficacy. Future studies focusing on the interplay between food intake, FAs and activities of their regulatory enzymes, and AN risk as well as prognosis would greatly improve our knowledge in the biological underpinning of AN, thereby contribute to development of more effective interventions and adjuvant therapies. 

## Figures and Tables

**Table 1:** Study subject characteristics.

Characteristic	All AN (*N* = 50)	IAN (*N* = 30)	RecAN (*N* = 20)	Controls (*N* = 47)	Statistics		
					All AN to Controls	IAN to Controls	RecAN to Controls
Age, year	30.1 ± 10.2	29.6 ± 9.0	31.1 ± 12.0	30.5 ± 9.3	0.710	0.68	0.88
BMI, kg/m^2^	18.9 ± 3.8	16.7 ± 2.4	22.3 ± 2.8	22.9 ± 3.5	<0.001 **	<0.001 **	0.5
BDI	21.8 ± 16.4	28.0 ± 16.7	12.6 ± 10.9	3.9 ± 7.4	<0.001 **	<0.001 **	<0.001 **
Fasting BAI	21.5 ± 13.7	26.6 ± 13.1	14.4 ± 11.6	4.3 ± 5.8	<0.001 **	<0.001 **	<0.001 **
Change in BAI	−3.4 ± 7.5	−5.1 ± 10.9	−2.3 ± 4.5	−1.3 ± 3.3	0.013 *	0.109	0.025 *

Note: Entries are of the form mean +/− SD. Statistical comparisons for controls versus each of the three AN groups (all AN, ill AN, and recovered AN) were tested using Wilcoxon rank sum tests. Statistics: * = 0.01 ≤ *p*-value < 0.05; ** = *p*-value < 0.01. BMI: body mass index; BDI: Becker Depression Inventory; BAI: Becker Anxiety Inventory. IAN: Ill anorexia nervosa with BMI ≤ 17.5; RecAN: recovered anorexia nervosa with BMI ≥ 18.5 for longer than one year.

**Table 2:** Fasting correlation coefficients of fatty acids with anorexia nervosa phenotypes.

Fatty Acids (All Subjects)	BMI		Fasting BDI		Fasting BAI		Change in BAI	
	*r*_s_	*p*-Value	*r*_s_	*p*-Value	*r*_s_	*p*-Value	*r*_s_	*p*-Value
Lauric acid (12:0 saturated)	−0.28	0.005	0.15	0.150	0.10	0.350	0.02	0.850
Alpha-linoleic acid (ALA) (18:3 *n*-3)	−0.46	<0.001	0.31	0.002	0.28	0.006	−0.04	0.744
Eicosapentaenoic acid (EPA) (20:5 *n*-3)	−0.29	0.004	0.28	0.005	0.26	0.010	−0.12	0.312
Docosapentaenoic acid (DPA) (22:5 *n*-3)	−0.39	<0.001	0.31	0.002	0.24	0.019	0.03	0.785
Fatty Acids (AN Group)								
							
Lauric acid (12:0 saturated)	−0.27	0.062	0.17	0.251	0.04	0.765	−0.15	0.446
Alpha-linoleic acid (ALA) (18:3 *n*-3)	−0.33	0.019	−0.03	0.832	−0.09	0.534	0.08	0.684
Eicosapentaenoic acid (EPA) (20:5 *n*-3)	−0.11	0.441	−0.02	0.900	−0.14	0.318	0.03	0.876
Docosapentaenoic acid (DPA) (22:5 *n*-3)	−0.30	0.032	0.27	0.060	0.05	0.727	0.05	0.782
Fatty Acids (Control Group)								
							
Lauric acid (12:0 saturated)	−0.15	0.318	−0.11	0.474	−0.15	0.326	0.14	0.347
Alpha-linoleic acid (ALA) (18:3 *n*-3)	−0.23	0.116	<0.01	0.980	−0.03	0.840	0.10	0.493
Eicosapentaenoic acid (EPA) (20:5 *n*-3)	−0.20	0.175	0.21	0.162	0.26	0.080	−0.10	0.498
Docosapentaenoic acid (DPA) (22:5 *n*-3)	−0.24	0.111	−0.06	0.690	−0.03	0.843	0.12	0.427

Note: Fasting **c**orrelation coefficients between fatty acids and phenotypes in all subjects combined, anorexia nervosa group, and control group. Correlation coefficients and p-values were calculated using Spearman’s correlation test. BMI: body mass index; BDI: Becker Depression Inventory; BAI: Becker Anxiety Inventory.

**Table 3:** Postprandial correlation coefficients of fatty acids with anorexia nervosa phenotypes.

Fatty Acids (All Subjects)	BMI		Fasting BDI		Postprandial BAI		Change in BAI	
	*r*_s_	*p*-Value	*r*_s_	*p*-Value	*r*_s_	*p*-Value	*r*_s_	*p*-Value
Lauric acid (12:0 saturated)	−0.27	0.018	0.06	0.640	0.10	0.408	0.06	0.592
Alpha-linoleic acid (ALA) (18:3 *n*-3)	−0.26	0.027	0.28	0.016	0.24	0.037	−0.25	0.030
Eicosapentaenoic acid (EPA) (20:5 *n*-3)	−0.15	0.191	0.25	0.033	0.28	0.017	−0.09	0.435
Docosapentaenoic acid (DPA) (22:5 *n*-3)	−0.17	0.198	0.15	0.198	0.16	0.183	−0.10	0.393
Fatty Acids (AN Group)								
							
Lauric acid (12:0 saturated)	−0.36	0.058	0.06	0.764	<0.01	0.99	0.05	0.812
Alpha-linoleic acid (ALA) (18:3 *n*-3)	−0.14	0.49	0.01	0.959	−0.08	0.702	−0.1	0.602
Eicosapentaenoic acid (EPA) (20:5 *n*-3)	0.12	0.544	0.09	0.648	−0.04	0.854	0.01	0.979
Docosapentaenoic acid (DPA) (22:5 *n*-3)	<0.01	0.982	0.14	0.48	0.03	0.866	−0.16	0.403
Fatty Acids (Control Group)								
							
Lauric acid (12:0 saturated)	−0.16	0.294	−0.04	0.806	0.05	0.746	0.11	0.477
Alpha-linoleic acid (ALA) (18:3 *n*-3)	−0.27	0.074	0.28	0.06	0.34	0.022	−0.14	0.358
Eicosapentaenoic acid (EPA) (20:5 *n*-3)	−0.22	0.135	0.23	0.119	0.33	0.026	−0.02	0.875
Docosapentaenoic acid (DPA) (22:5 *n*-3)	−0.21	0.157	0.09	0.545	0.15	0.332	0.01	0.941

Note: Postprandial correlation coefficients between fatty acids and phenotypes in all subjects combined, anorexia nervosa group, and control group. Correlation coefficients and p-values were calculated using Spearman’s correlation test. BMI: body mass index; BDI: Becker Depression Inventory; BAI: Becker Anxiety Inventory.
